# *Xenopus borealis* as an alternative source of oocytes for biophysical and pharmacological studies of neuronal ion channels

**DOI:** 10.1038/srep14763

**Published:** 2015-10-06

**Authors:** Ben Cristofori-Armstrong, Ming S. Soh, Sahil Talwar, Darren L. Brown, John D. O. Griffin, Zoltan Dekan, Jennifer L. Stow, Glenn F. King, Joseph W. Lynch, Lachlan D. Rash

**Affiliations:** 1 Institute for Molecular Bioscience, The University of Queensland, St Lucia, QLD 4072, Australia; 2 Queensland Brain Institute, The University of Queensland, St Lucia, QLD 4072, Australia; 3 School of Biomedical Sciences, The University of Queensland, St Lucia, QLD 4072, Australia

## Abstract

For the past 30 years, oocytes from *Xenopus laevis* have been extensively used to express and characterise ion channels in an easily controlled environment. Here we report the first use of oocytes from the closely related species *Xenopus borealis* as an alternative expression system for neuronal ion channels. Using the two-electrode voltage-clamp technique, we show that a wide variety of voltage- and ligand-gated ion channels have the same channel properties and pharmacological profiles when expressed in either *X. laevis* or *X. borealis* oocytes. Potential advantages of the *X. borealis* oocytes include a smaller endogenous chloride current and the ability to produce more intense fluorescence signals when studied with voltage-clamp fluorometry. Scanning electron microscopy revealed a difference in vitelline membrane structure between the two species, which may be related to the discrepancy in fluorescence signals observed. We demonstrate that *X. borealis* oocytes are a viable heterologous system for expression of neuronal ion channels with some potential advantages over *X. laevis* oocytes for certain applications.

*Xenopus* frogs have been extensively used for almost a century as model organisms for a wide range of biological applications. *Xenopus* have been most extensively used for developmental and genetic studies, as external development of the embryos and their large size allows high throughput studies on a vertebrate system. Gurdon and colleagues were the first to show that injection of heterologous mRNA into *Xenopus laevis* oocytes resulted in robust expression of exogenous proteins^1^. Furthermore, *Xenopus* oocytes possess much of the cellular machinery required to produce functionally important post-translational modifications. These properties were exploited to show that *Xenopus* oocytes could faithfully produce surface expressed, functional nicotinic acetylcholine receptors following injection of mRNA from *Torpedo marmorata*^2^. *Xenopus* oocytes subsequently became a valuable tool for the study of mammalian membrane proteins, receptors, and ion channels particularly in the fields of neuroscience and pharmacology^3^,^4^. With the growing number of genomic studies identifying mutations associated with human diseases, the oocyte system continues to be of importance in determining the functional effect of these mutations in expressed proteins^5^,^6^, and as a tool to screen for and characterise novel modulators of ion channels or receptors known to be involved in poorly treated conditions such as pain^7^,^8^.

There are more than 20 species of *Xenopus*, but the African clawed frog *X. laevis* appears to be the only species that has been used for electrophysiological studies of ion channels. *X. tropicalis* and the Marsabit clawed frog *X. borealis* are widely used in many laboratories for genetic and developmental studies^9^,^10^,^11^,^12^,^13^,^14^. Although *X. tropicalis* is more widely used, *X. borealis* is more closely related to *X. laevis*^15^. However, heterologous ion channel expression has yet to be performed using *X. borealis* oocytes.

Here we present data showing the first use of *X. borealis* oocytes for electrophysiology studies, and provide a comparison with *X. laevis* oocytes. We analysed the expression profile of a variety of voltage- and ligand-gated ion channels using two-electrode voltage-clamp and voltage-clamp fluorometry (VCF) methods. Furthermore, we investigated the vitelline and plasma membrane of *X. laevis* and *X. borealis* oocytes using scanning electron microscopy (SEM), and observed a clear difference in membrane structure. We found that *X. borealis* oocytes can be successfully used as a heterologous expression system for neuronal ion channel studies.

## Results

### Comparison of *X. laevis* and *X. borealis* oocytes

**Figure 1:**
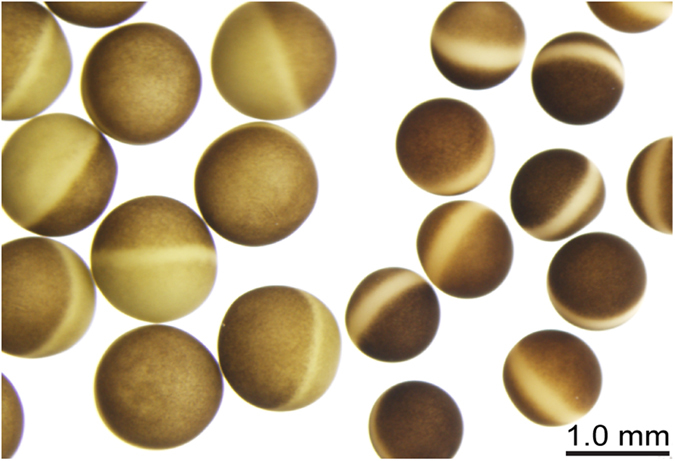
Comparison of defolliculated stage V–VI oocytes from *X. laevis* (left) and *X. borealis* (right). Oocytes were imaged on an Olympus SZX12 stereomicroscope in ND96 solution.

*X. laevis* and *X. borealis* oocytes can be easily distinguished visually by their size and the pigmentation of their vegetal hemispheres (Fig. 1). Stage V–VI oocytes from *X. laevis* (1.0–1.3 mm diameter) have a pale yellow (sometimes pale green) vegetal hemisphere. *X. borealis* oocytes are smaller (0.8–1.1 mm diameter), have a darker animal hemisphere and brown vegetal hemisphere clearly separated by a light-yellow belt around the middle. The resting membrane potential of healthy oocytes expressing ion channels of interest from both species was approximately –35 mV over the period in which electrophysiology recordings were performed (with the exception of voltage-gated potassium (K_V_) channels that had a more negative resting membrane potential around –60 mV). The microinjection process also revealed that the defolliculated membranes of *X. borealis* oocytes were stronger and more resistant to injection than those of *X. laevis*, suggesting a difference in the vitelline membrane (see below).

**Figure 2:**
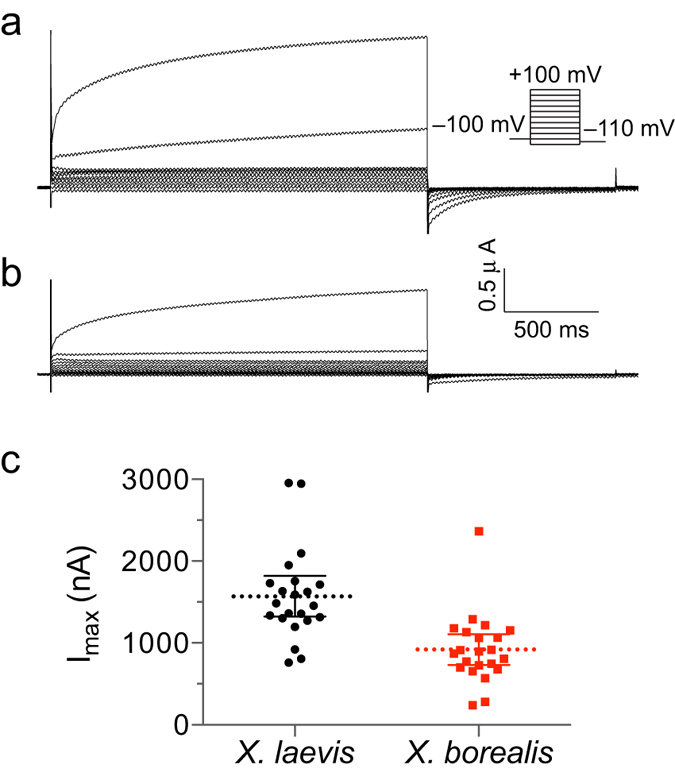
Endogenous Ca^2+^ -activated chloride currents in naïve *X. laevis* (•) and *X. borealis* (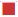) oocytes. Representative families of whole-cell currents elicited by steps from −120 mV to +100 mV in 20-mV increments in oocytes from (**a**) *X. laevis* and (**b**) *X. borealis* (holding potential of −100 mV). (**c**) Peak amplitude as measured at the end of a 2 s pulse to +100 mV. A significant difference was observed between the two species (unpaired t-test, *P* = 8.3 × 10^−5^). Each data point indicates recording from a single oocyte (*n* = 22). Several oocytes were used from each of five individual frogs of each species. Data are presented as mean (dashed line) and 95% confidence intervals.

One of the main advantages of the *X. laevis* expression system for ion channel studies is the low level of endogenously expressed channels and receptors, which yields a relatively electrophysiologically silent background. Nevertheless, the most prominent endogenous current in these cells is from the well-characterised Ca^2+^ -activated chloride channel (Fig. 2a)^16^,^17^. This outward current was also observed in *X. borealis* oocytes when subject to high voltage pulses (greater than +80 mV) (Fig. 2b). As has been observed for *X. laevis* oocytes, the amplitude of this current in *X. borealis* was batch dependent and present in ~85% of all oocytes tested. However, the average amplitude of this current was significantly smaller in *X. borealis* oocytes (Fig. 2c). No other protocols used in this study to activate the ion channels tested (voltage- or ligand-gated) led to the generation of observable currents in naïve oocytes from either *X. laevis* or *X. borealis*.

### Voltage-gated ion channels

**Figure 3:**
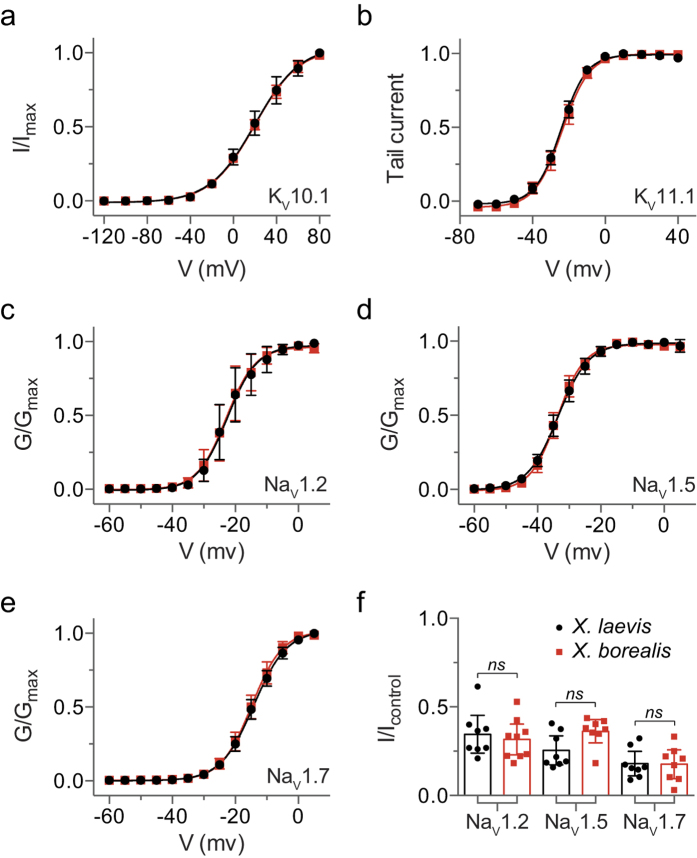
Voltage-dependent properties of K_V_ and Na_V_ channels expressed in *X. laevis* (•) and *X. borealis* (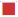) oocytes. (**a**) Current-voltage relationship showing activation of K_V_10.1 channels (*n* = 12). (**b**) K_V_11.1 tail current plotted as a function of voltage to show activation properties (*n* = 9–12). The normalised deduced conductance (G)-voltage relationships for (**c**) Na_V_1.2, (**d**) Na_V_1.5, and (**e**) Na_V_1.7 (*n* = 12–17). (**f**) The effect of 300 nM ProTx-I on current evoked by a depolarisation to –15 mV from a holding potential of −80 mV (*n* = 8–9). There is no evidence of a difference between the two species (unpaired t-test, *P* > 0.05). Error bars indicate 95% confidence intervals.

Two-electrode voltage-clamp electrophysiology recordings were performed to compare the properties of a panel of K_V_ and voltage-gated sodium (Na_V_) channels heterologously expressed in *X. laevis* and *X. borealis* oocytes. The voltage sensitivity of K_V_10.1 (hEAG) activation (Fig. 3a) and time constants of deactivation (Supplementary Fig. S1 and Table S1) were comparable between species. Likewise, the threshold of activation in K_V_11.1 (hERG) did not differ between species (Fig. 3b). Conductance-voltage relationship curves obtained for Na_V_1.2 (Fig. 3c), Na_V_1.5 (Fig. 3d), and Na_V_1.7 (Fig. 3e) overlap well between *X. laevis* and *X. borealis* oocytes. Pharmacological modulation of Na_V_ channels was assessed by comparing the inhibition induced by 300 nM ProTx-I, a potent and non-selective Na_V_ channel inhibitor^18^. Comparable levels of inhibition between species were detected for all Na_V_ channel subtypes tested (Fig. 3f). There is no evidence of a difference in current amplitudes for voltage-gated ion channels expressed in *X. laevis* as compared to *X. borealis* (Table 1).

### Ligand-gated ion channels

**Figure 4:**
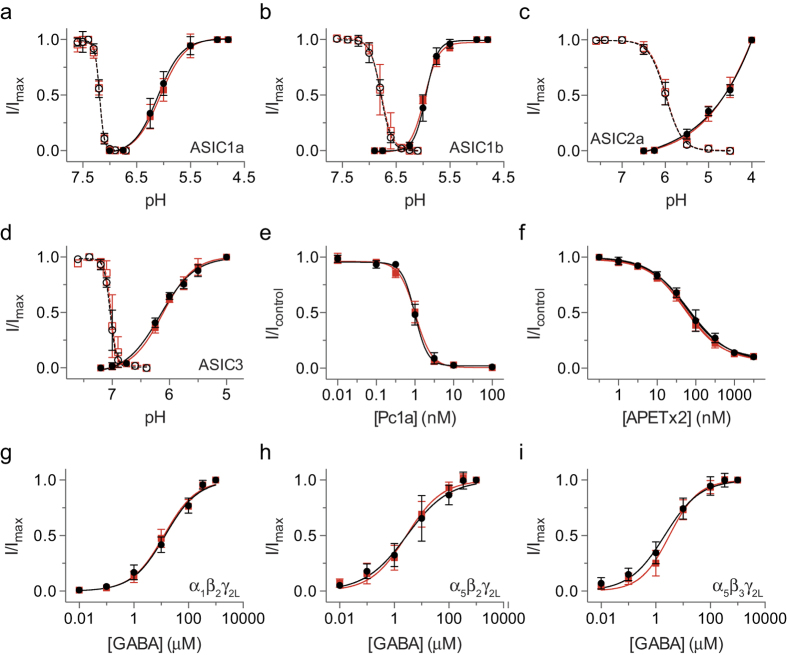
The effect of activating and antagonist ligands on different ASIC and GABA_A_R subtypes expressed in *X. laevis* (•) and *X. borealis* (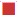) oocytes. pH-dependence of steady-state desensitisation (open symbols, dashed lines) and activation (closed symbols, solid lines) of (**a**) ASIC1a, (**b**) ASIC1b, (**c**) ASIC2a, and (**d**) ASIC3 (*n* = 9–12). (**e**) Concentration-effect curve for inhibition of rat ASIC1a by Pc1a (*n* = 9–12). (**f**) Concentration-effect curve for inhibition of rat ASIC3 by APETx2 (*n* = 9). Concentration-effect curves for GABA activation of the (**g**) *α*_1_*β*_2_*γ*_2L_, (**h**) *α*_5_*β*_2_*γ*_2L_, and (**i**) *α*_5_*β*_3_*γ*_2L_ subtypes of GABA_A_Rs (*n* = 9–10). Error bars indicate 95% confidence intervals.

The two-electrode voltage-clamp method was also applied to a range of ligand-gated ion channels expressed in *X. laevis* and *X. borealis* oocytes. The pH-sensitivity of activation and steady-state desensitisation for homomeric acid-sensing ion channels (ASICs) were similar between species for ASIC1a (Fig. 4a), ASIC1b (Fig. 4b), ASIC2a (Fig. 4c), and ASIC3 (Fig. 4d). To assess any differences in the pharmacological properties of ASICs expressed in oocytes from each species, the activity of two known peptide inhibitors of ASICs was tested. π-TRTX-Pc1a (Pc1a, also known as PcTx1) inhibited rat ASIC1a expressed in both *X. laevis* and *X. borealis* oocytes with an IC_50_ of 1 nM (Fig. 4e), which corresponds well with the reported literature value of 0.9 nM obtained using *X. laevis* oocytes^19^. APETx2 inhibited rat ASIC3 expressed in *X. laevis* and *X. borealis* oocytes with an IC_50_ of 65 nM and 52 nM (Fig. 4f), in good agreement with the reported value of 61 nM for APETx2 in *X. laevis* oocytes^20^. We also found no difference in the concentration-effect curves for GABA activation of the *α*_1_*β*_2_*γ*_2L_ (Fig. 4g), *α*_5_*β*_2_*γ*_2L_ (Fig. 4h), and *α*_5_*β*_3_*γ*_2L_ (Fig. 4i) subtypes of *γ*-aminobutyric acid type A receptor (GABA_A_Rs) when expressed in *X. laevis* and *X. borealis* oocytes. There is no evidence of a difference in current amplitudes for ligand-gated ion channels expressed in *X. laevis* as compared to *X. borealis* (Table 1).

### Voltage-clamp fluorometry

**Figure 5:**
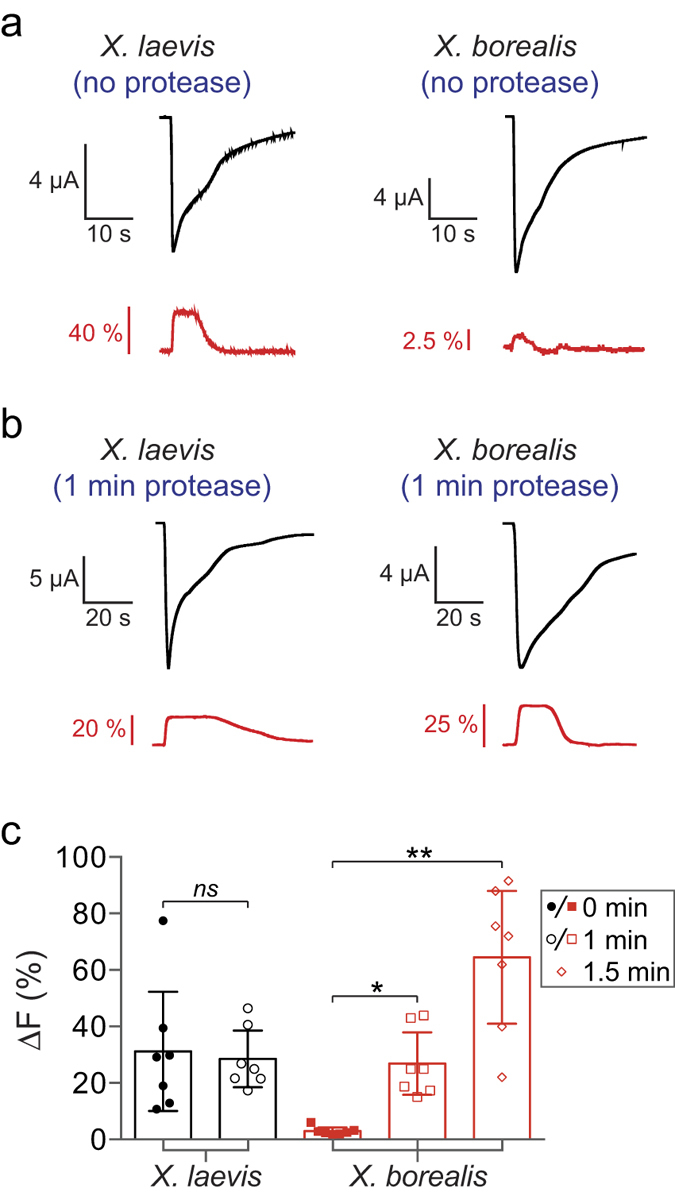
Effect of protease treatment on fluorescence signals (Δ*F*) from *X. laevis* and *X. borealis* oocytes expressing the *α*_1_N203C GlyR with current induced by 10 μM glycine. (**a**) Representative current and fluorescence traces show a significantly greater *ΔF* in untreated *X. laevis* than *X. borealis* oocytes. (**b**) A significant increase in *ΔF* was obtained after 1 min treatment with protease for *X. borealis*, but not *X. laevis,* oocytes. Traces from A and B represent separate oocytes. (**c**) ΔF of *X. laevis* and *X. borealis* oocytes in all conditions tested (*n* = 7). *X. laevis* oocytes were not amenable to protease treatment for >1 min as this damaged membrane integrity. *P* values were calculated in comparison to untreated oocytes from each species using an unpaired t-test with Welch’s correction (*P < 0.01, **P < 0.001). Error bars indicate 95% confidence intervals.

VCF enables study of the conformational rearrangements undertaken by ion channels during gating. *X. laevis* oocytes are the most commonly used expression system for VCF recordings^21^. Although expression of the *α*_1_N203C glycine receptor (GlyR) was confirmed in *X. laevis* and *X. borealis* oocytes, a glycine-induced fluorescence change (*ΔF*) signal was only observed in *X. laevis* (Fig. 5a). Considering the difference in oocyte vitelline membrane strength between the two species observed upon injecting cRNA, we hypothesised that a difference in this membrane may account for the lack of *ΔF* responses in *X. borealis*. To test this hypothesis, oocytes were subjected to protease treatment to disrupt the vitelline membrane. Protease treatment was used to replace manual removal of the vitelline membrane by forceps for this experiment, as we found complete removal rendered the oocytes extremely fragile and vulnerable to rupture.

**Figure 6:**
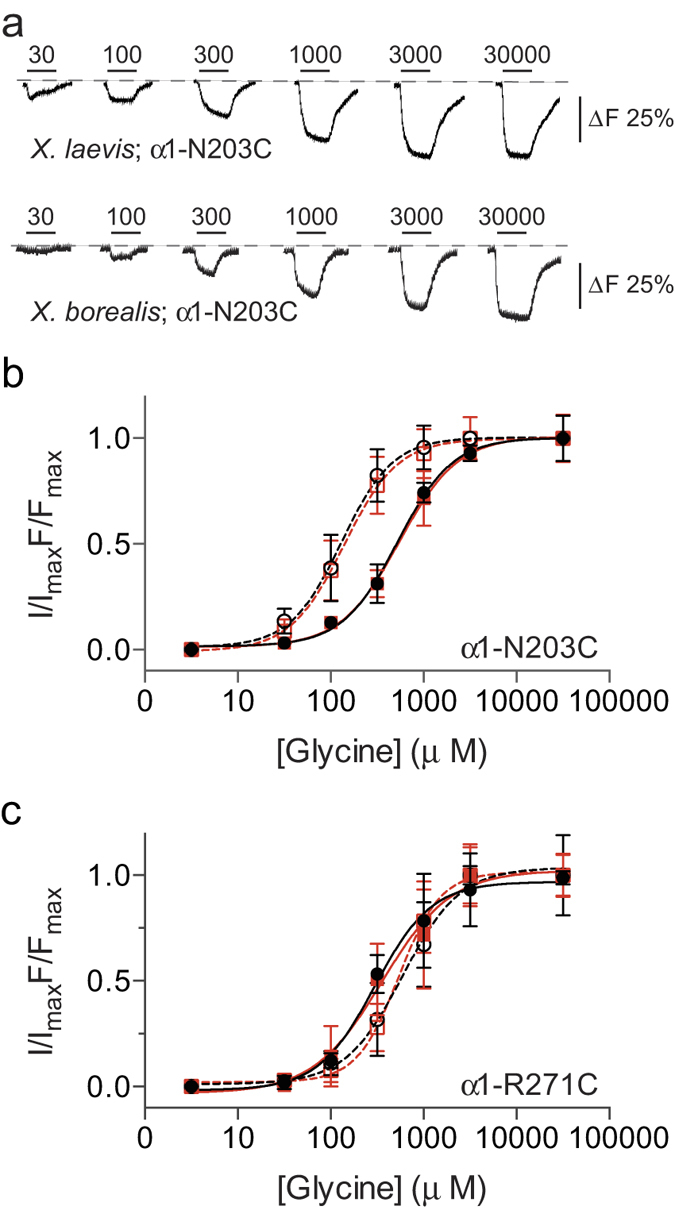
Expression of GlyRs in *X. laevis* (•) and *X. borealis* (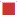) oocytes as determined by voltage-clamp fluorometry. (**a**) Example *ΔF* traces from oocytes expressing labelled *α*_1_N203C GlyR. (**b**) and (**c**) Normalised glycine concentration-effect curves for both current (closed symbols, solid lines) and fluorescence (open symbols, dashed lines) of MTS-TAMRA labelled *α*_1_N203C GlyR and MTSR labelled *α*_1_R271C GlyR using voltage-clamp fluorometry (*n* = 4–6). Error bars indicate 95% confidence intervals.

Protease treatment of *X. borealis* oocytes prior to labelling substantially improved *ΔF* responses (Fig. 5b). This was observed in a time-dependent manner, with a positive correlation between increased *ΔF* and protease incubation time (Fig. 5c). In contrast, protease treatment of *X. laevis* oocytes for 1 min did not increase the *ΔF* (Fig. 5c). Indeed, *X. laevis* oocytes treated for >1 min became very fragile and did not survive impalement by electrodes for VCF recordings. We further investigated the glycine-induced *ΔF* responses and quantified the glycine-induced current (*ΔI*) and *ΔF* concentration-effect relationships for labelled *α*_1_N203C (Fig. 6a,b) and *α*_1_R271C GlyRs (Fig. 6c). Both mutants had similar EC_50_, slope, *ΔI*_max_ and *ΔF*_max_ values in *X. laevis* and *X. borealis* oocytes (summarised in Supplementary Table S2).

**Figure 7:**
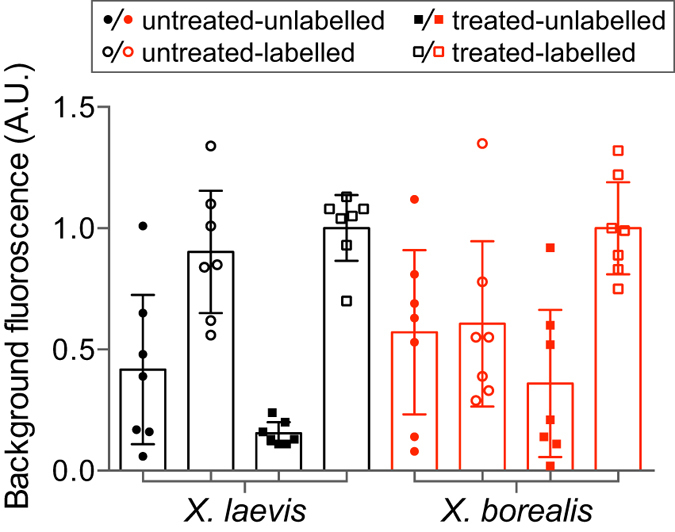
Protease treatment affects dye accessibility to channels in the plasma membrane. Effect of MTS-TAMARA labelling on the background fluorescence of *X. laevis* (black) and *X. borealis* (red) oocytes expressing *α*_1_N203C GlyR prior to (untreated, circles) and following protease exposure (1 min treated, squares) (*n* = 7). Fluorescence readings were normalised to oocytes that had been protease treated then labelled. Error bars indicate 95% confidence intervals.

In order to test if the fluorescent dyes were simply not penetrating the vitelline membrane of *X. borealis* or if the membrane was somehow binding the dye, the level of background fluorescence was measured for oocytes from both species under a variety of conditions. The background fluorescence of *X. laevis* oocytes increased to a similar degree upon sulfo rhodamine methanethiosulfonate (MTSR) labelling irrespective of protease treatment. In contrast, the background fluorescence of *X. borealis* oocytes only increased following protease treatment then labelling (Fig. 7).

### Scanning electron microscopy

**Figure 8:**
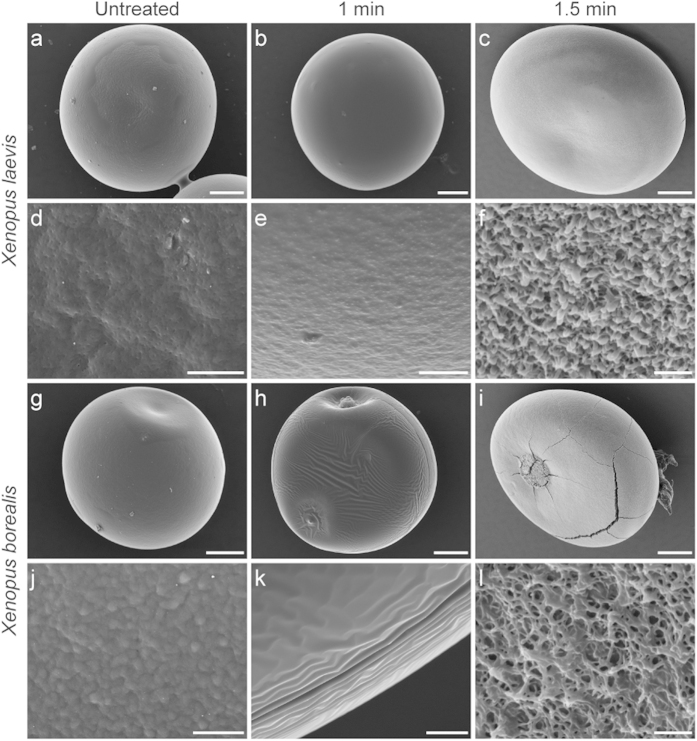
SEM images of defolliculated *X. laevis* and *X. borealis* stage V–VI oocytes prior to and following protease treatment. Oocytes from *X. laevis* (**a**–**f**) and *X. borealis* (**g**–**l**) were viewed by SEM following treatment with protease for 1 min (**b**,**e**,**h**,**k**) and 1.5 mins (**c**,**f**,**i**,**l**). At higher magnifications the typically contoured surface and pores of the vitelline membrane of untreated *X. laevis* (**d**) and *X. borealis* (**j**) become apparent, scale bar = 10 μm. One minute protease treatment of oocytes affects the vitelline membrane of *X. laevis* (**e**) and *X. borealis* (**k**) differently, scale bar = 10 μm. Microvilli projections on the plasma membranes of *X. laevis* (**f**) and *X. borealis* (**l**) oocytes show different morphology and density, scale bar = 2 μm. Each panel represents a separate oocyte.

It was apparent that *X. laevis* and *X. borealis* oocytes have distinct differences in their vitelline membrane properties. To gain further insights into this difference, the oocyte surfaces were imaged using scanning electron microscopy (SEM) at each time point during the protease treatment procedure. *X. laevis* oocytes without protease treatment (Fig. 8a,d) and after 1 min treatment (Fig. 8b,e) were very similar. In contrast, treatment for 1.5 min (Fig. 8c,f) completely removed the vitelline membrane and exposed the underlying plasma membrane. At low magnification, untreated *X. borealis* oocytes (Fig. 8g) largely resemble untreated *X. laevis* oocytes. However, protease treatment for 1 min results in a clearly wrinkled vitelline membrane (Fig. 8h,k) and after 1.5 min of treatment the plasma membrane is exposed (Fig. 8i,l).

The higher magnification images more clearly revealed differences in the membranes of *X. laevis* and *X. borealis* oocytes. The vitelline membrane of *X. laevis* (Fig. 8d) appears to be smoother yet more porous when compared to that of *X. borealis* (Fig. 8j), as evidenced by the uniform distribution of pores. There was also a notable difference between species in the effect of protease on the vitelline membrane. The membrane of *X. laevis* appears largely unaffected after 1 min exposure to protease (Fig. 8e), whereas the integrity of the *X. borealis* membrane is obviously compromised and showed substantial wrinkling (Fig. 8k). The structure and distribution of microvilli projections on *X. laevis* (Fig. 8f) and *X. borealis* (Fig. 8l) plasma membranes are also quite distinct. As previously imaged, the *X. laevis* microvilli show a flattened surface and appear tightly compressed^22^,^23^. In contrast, the *X. borealis* microvilli are more filamentous and sparsely packed, resulting in what appears to be a more porous plasma membrane structure.

## Discussion

We report here the first use of *X. borealis* oocytes for the functional characterisation of neuronal ion channels, and identify some potential advantages over the classic *X. laevis* oocyte expression system. Voltage- and ligand-sensitivity, as well as exogenous channel expression levels, were very similar between the two species and comparable to literature values for all ion channels tested (summarised in Supplementary Tables S2, S3). The pharmacological profiles of channels expressed in oocytes from each species were essentially identical in terms of efficacy and potency of small molecules agonists to larger peptide antagonists. Our results reveal that oocytes from *X. borealis* are a reliable substitute for *X. laevis* oocytes for functional and pharmacological characterisation of ion channels.

Interestingly, we observed reduced maximal current levels of the endogenous Ca^2+^ -activated chloride channel in *X. borealis* oocytes. This endogenous current is one of the biggest drawbacks of studying voltage-gated ion channels in *X. laevis* oocytes^24^. The current is observed when using high-voltage pulses and can interfere with interpretation of electrophysiological recordings particularly when studying current-voltage relationships, or channels that are substantially permeable to Ca^2+^. Considering an equivalent level of endogenous channel density across the oocyte membrane of both species, it is expected that the smaller *X. borealis* oocytes would have a smaller total Ca^2+^ -activated chloride channel current. However we show that overexpression of exogenous channels is similar between species (Table 1), and therefore the ratio of exogenous to endogenous current levels would nevertheless be greater for *X. borealis* oocytes. Thus, the significantly smaller endogenous current size in *X. borealis* (Fig. 2) provides an unexpected advantage over the commonly used *X. laevis* oocytes.

Additionally, VCF analysis highlighted an electrophysiologically important difference between oocytes from the two species. Despite the initial lack of fluorescence signal in *X. borealis* oocytes, we devised a protease treatment protocol that resulted in an average signal much greater than that observed from *X. laevis* oocytes (Fig. 5). This significant improvement in fluorescence signals has the potential to facilitate VCF recordings from proteins or specific channel mutants that have previously suffered from poor fluorescence signals.

The increase in fluorescence signal observed after incubating *X. borealis* oocytes in protease prior to labelling suggests that either (i) the vitelline membrane is selectively impermeable to the dyes used or that (ii) the dye is somehow sequestered by the *X. borealis* membrane before it can access the channels in the plasma membrane. If the second option were true, we would expect to see a large increase in the background fluorescence of *X. borealis* oocytes following incubation with the dye prior to protease treatment. However, we showed that this is not the case, and that the dye appears able to access the free cysteine on the GlyR mutant only once the vitelline membrane has been compromised by the action of protease, as we could see using SEM imaging. This suggests that the vitelline membrane of *X. borealis* is somehow selectively impermeable to the thiol containing rhodamine dyes. Several ligands (ranging in size from protons to 4.5 kDa peptides) of both ligand- and voltage-gated ion channels studied were able to freely cross the vitelline membrane and have an equal action in the same time course on oocytes from both *X. laevis* and *X. borealis*. All the ligands used in this study that show no difference in activity between the species have a net positive charge at physiological pH, whereas MTSR and MTS-TAMARA are both neutral at pH 7. It could be that the vitelline membrane of *X. borealis* has unique properties that impede the permeability of neutral or negatively charged molecules. We are currently investigating this possibility further. Imaging of the surface morphology of oocytes from both species highlighted a substantial difference in the structure of the plasma membrane. We noted a dramatic increase in the fragility of the *X. laevis* oocytes after vitelline membrane removal as compared to the oocytes from *X. borealis* and suggest that the difference in plasma membrane structure could account for this observation.

Although *X. laevis* is the most widely used source of oocytes for ion channel studies, the oocytes of several other species have also been assessed for their suitability as a heterologous expression system for membrane proteins. Functional expression of the eel electroplax acetylcholine receptor was demonstrated in oocytes from the Japanese fire belly newt, *Cynops pyrrhogaster*^25^. Despite the larger size (1.6–1.9 mm in diameter) and apparently more robust nature of *Cynops* oocytes as compared to *X. laevis* oocytes^26^, this system does not appear to have been widely adopted for ion channel studies, likely due to limited availability. The cane toad, *Bufo marinus*, has also been assessed as a possible substitute source of oocytes, and was shown to satisfactorily express K_V_1.1 channels^27^. However, the levels of endogenous background current were slightly higher in *B. marinus* oocytes than those from *X. laevis*. This finding may explain the observation that oocytes from *B. marinus* have quite leaky membranes^28^. Furthermore, *B. marinus* oocytes were less effective than those from *X. laevis* at expressing human amino acid transporters^28^. In contrast, oocytes from the axolotl (or Mexican salamander, *Ambystoma mexicanum*) appear to have lower levels of endogenous ion channels. In particular they lack the Ca^2+^ -activated chloride channel, a property that was exploited to aid expression cloning of this channel from *Xenopus* oocytes and characterisation of mouse orthologues^29^. Despite the promising discovery of an oocyte with an electrically cleaner background, *Ambystoma* oocytes have not become a commonly used model system.

In conclusion, we demonstrated that oocytes from *X. borealis* are an excellent model system for expressing exogenous ion channels for electrophysiological and fluorescence studies. We also show that *X. borealis* oocytes have a reduced background chloride current, and have the potential to produce a larger fluorescence signal for VCF recordings after protease treatment. This is of increasing importance due to the growing number of mutant channels being identified as influential in several human diseases that need be better characterised.

## Methods

### Two-electrode voltage clamp of *Xenopus* oocytes

*X. laevis* (Xenopus Express) and *X. borealis* (Nasco) oocytes were surgically removed from anaesthetised (Tricaine methanesulfonate, MS-222) female frogs and treated with collagenase (1 mg/ml; Sigma type I) for defolliculation. cRNAs were synthesised using an mMessage mMachine cRNA transcription kit (Ambion Inc., Austin, TX, USA) and injected into stage V–VI oocytes at 4–200 ng per cell. Oocytes were stored at 17 °C in ND96 solution (96 mM NaCl, 1.8 mM CaCl_2_, 2 mM KCl, 2 mM MgCl_2_, 5 mM HEPES, pH 7.4) supplemented with 2.5 mM pyruvic acid, 50 μg/mL gentamicin, and either 2.5% horse serum or 0.5 mM theophylline. Membrane currents were recorded 2–10 days after injection under voltage-clamp (Axoclamp 900A or Geneclamp 500B, Molecular Devices, CA, USA) using two standard glass microelectrodes of 0.5–2 MΩ resistance when filled with 3 M KCl solution. Data acquisition and analysis were performed using pCLAMP software (Version 9.2 or 10, Molecular Devices, Sunnyvale, CA, USA). All experiments were performed at room temperature (18–21 °C) in ND96 solution. Experiments containing peptides were performed in ND96 solution containing 0.1% fatty acid free-bovine serum albumin (Sigma). Recordings were performed as previously described for ASICs^30^,^31^, GABA_A_R^32^,^33^, GlyR^34^, K_V_^6^,^8^,^35^, and Na_V_^36^ channels.

### Ethics Statement

This study was carried out in strict accordance with the recommendations in the Australian code of practice for the care and use of animals for scientific purposes, 8^th^ Ed. 2013. The protocol was approved by the Anatomical Biosciences group of the Animal Ethics Committee at The University of Queensland (Approval Number: QBI/059/13/ARC/NHMRC). All surgery was performed under anaesthesia (animals bathed in 1.3 mg/ml of MS-222), and all efforts were made to minimise suffering.

### Voltage-clamp fluorometry

This technique permits the real time monitoring of conformational changes in ion channel domains distant from the gate. It involves introducing a cysteine into the ion channel domain of interest and covalently labelling it with a sulfhydryl-reactive rhodamine derivative such as MTSR. As the quantum efficiency of the rhodamine depends on the hydrophobicity of its environment, protein conformational changes occurring in the immediate vicinity of the attached fluorophore are likely to change the hydrophobicity of its environment and thus produce a fluorescence change^21^. Hence, by monitoring oocyte fluorescence intensity, conformational changes in domains distant from the channel gate can be monitored in real time. Conformational changes at the channel gate are monitored simultaneously using two-electrode voltage-clamp. For these recordings, DNA encoding the *α*1 GlyR subunit was subcloned into pGEMHE, a plasmid vector optimised for oocyte expression. Two residues were chosen for fluorophore labelling: N203C in loop C of the glycine binding site, and R271C at the external end of the TM2 domain. Both sites have previously been characterised extensively using voltage-clamp fluorometry^34^,^37^,^38^. A C41A mutation was incorporated into the *α*1 GlyR to eliminate the sole non-crosslinked extracellular sulfhydryl group. Site directed mutagenesis was performed using the QuikChange mutagenesis kit (Stratagene). Successful incorporation of the N203C and R271C mutations were confirmed through automated sequencing of the entire cDNA-coding region.

On the day of recording, oocytes were transferred into ND96 and stored on ice. For a subset of experiments, a modified version of the previously described approach^39^ for vitelline membrane disruption and removal was performed by incubating oocytes in ND96 containing 0.5 mg/ml protease (protease type VIII from *Bacillus licheniformis*, Sigma-Aldrich) for 1–1.5 min before labelling. Following this, a sequential wash step was performed by transferring oocytes into fresh ND96 solution five consecutive times. To label with either MTSR or MTS-TAMRA (Toronto Research Chemicals), oocytes were transferred into labelling solution containing 10 μM of either compound in ND96 for 25 s. The oocytes were then washed and stored in ND96 for up to 6 h before recording. All labelling steps were performed on ice.

We recently described the experimental set up in detail^34^. Briefly, we employed an inverted microscope (Eclipse TE300, Nikon Instruments) equipped with a high-Q tetramethylrhodamine isothiocyanate filter set (Chroma Technology), a Plan Fluor X40 objective (Nikon Instruments) and an H7360-03 photomultiplier detection system (Hamamatsu Photonics) attached to the side port of the microscope. An excitation filter wheel including a shutter and emission filter wheel were controlled through a Lambda 10-2 unit (Sutter Instruments). A Lambda LS 175 W xenon arc lamp served as a light source and was coupled to the microscope via a liquid light guide (Sutter Instruments). The design of the custom-made recording chamber has been described^40^. An automated perfusion system operated by a ValveBank-8 valve controller (AutoMate Scientific) was used for perfusion of the recording chamber. Electrodes were moved by automated ROE-200 micromanipulators coupled to an MPC-200 controller (Sutter Instruments). Cells were voltage-clamped at −40 mV and currents recorded using a Gene Clamp 500B amplifier (Molecular Devices). Current and fluorescence traces were acquired at 200 Hz via a Digidata 1322A interface and pCLAMP 9.2 software.

### Peptide preparation

Recombinant APETx2 and Pc1a were produced as previously described^30^,^41^. ProTx-I was synthesised using Fmoc chemistry as described^42^.

### Data analysis and statistics

All data were analysed using Prism 6.0 (GraphPad Software, San Diego, CA, USA). The Hill equation was fitted to normalised concentration-response curves to obtain the half-maximal response (EC_50_, IC_50_, or pH_50_) and Hill coefficient (*n*H). Voltage-dependent activation curves were fit with the Boltzmann equation to obtain the V_0.5_ (the voltage corresponding to half-maximal effect). Data are shown as mean and 95% confidence intervals, and *n* represents the number of oocytes for a given experiment (taken from at least three different frogs). Most equivalence testing comparing *X. laevis* and *X. borealis* oocytes was performed using an unpaired Student’s *t*-test with 95% confidence intervals. A Welch’s *t*-test was performed when two populations were not assumed to have equal standard deviations (see Fig. 5). For both *t*-tests, it was assumed data was sampled from Gaussian populations, and *P* values were calculated from two-tailed tests. *P* < 0.05 was considered statistically significant, unless stated otherwise.

### Scanning electron microscopy

Oocytes were fixed in 2.5% glutaraldehyde in Modified Barth’s Solution (88 mM NaCl, 1 mM CaCl_2_, 1 mM KCl, 1 mM MgSO_4_, 2.5 mM NaHCO_3_, 5 mM HEPES, pH 7.6) at 4 °C for 12 h. Oocytes were then post fixed in 1% osmium tetroxide for 1 h, dehydrated through ethanols and finally dried using hexamethyldisilazane (HMDS) (all from ProSciTech, Queensland, Australia), before being coated with gold. Images were captured using a JEOL JCM5000 Neoscope scanning electron microscope (Laboratory Scientific Engineering).

## Additional Information

**How to cite this article**: Cristofori-Armstrong, B. *et al*. *Xenopus borealis* as an alternative source of oocytes for biophysical and pharmacological studies of neuronal ion channels. *Sci. Rep.* **5**, 14763; doi: 10.1038/srep14763 (2015).

## Supplementary Material

Supplementary Information

## Figures and Tables

**Table 1:** Maximal current amplitudes recorded from exogenous channels expressed in *X. laevis* and *X. borealis* oocytes.

**Ion Channel**	**Maximal current (μA)**		***P*** **value**	***n*** **number**
	***X. laevis***	***X. borealis***		
K_V_10.1	21.0 ± 11.8	23.6 ± 8.5	0.49	15
K_V_11.1	1.1 ± 0.8	0.9 ± 0.2	0.31	14
Na_V_1.2	5.3 ± 3.6	8.0 ± 5.3	0.16	11
Na_V_1.5	3.3 ± 3.0	5.1 ± 2.5	0.08	15
Na_V_1.7	2.2 ± 1.4	1.9 ± 0.5	0.38	12
ASIC1a	4.8 ± 4.5	3.9 ± 3.0	0.44	20
ASIC1b	3.5 ± 2.9	5.5 ± 4.2	0.13	20
ASIC2a	3.8 ± 3.0	3.8 ± 3.7	0.96	18
ASIC3	1.2 ± 1.1	1.3 ± 0.8	0.94	23
GABA_A_ *α*_1_*β*_2_*γ*_2L_	7.0 ± 2.3	7.3 ± 2.7	0.77	9
GABA_A_ *α*_5_*β*_2_*γ*_2L_	3.5 ± 1.7	4.4 ± 2.2	0.35	10
GABA_A_ *α*_5_*β*_3_*γ*_2L_	3.8 ± 2.0	3.9 ± 2.6	0.89	7
GlyR *α*_1_N203C	8.4 ± 0.5	8.0 ± 2.0	0.63	6
GlyR *α*_1_R271C	3.4 ± 4.2	2.7 ± 1.2	0.69	6

Maximal current ± s.d. *P* values were calculated comparing *X. laevis* and *X. borealis* using an unpaired *t*-test.

## References

[b1] Gurdon J. B., Lane C. D., Woodland H. R. & Marbaix G. Use of frog eggs and oocytes for the study of messenger RNA and its translation in living cells. Nature 233, 177–182 (1971). 4939175 10.1038/233177a0

[b2] Barnard E. A., Miledi R. & Sumikawa K. Translation of exogenous messenger RNA coding for nicotinic acetylcholine receptors produces functional receptors in *Xenopus* oocytes. Proc. R. Soc. Lond. B Biol. Sci . 215, 241–246 (1982). 6127706 10.1098/rspb.1982.0040

[b3] Goldin A. L. in *Expres*sion a*nd* anal*ysis of recombinant ion channels: From Structural Studies to Pharmacological Screening* (ed Clare J. J., Treise D. J. ) (Wiley-VCH Verlag GmbH & Co., 2006).

[b4] Sigel E. Use of *Xenopus* oocytes for the functional expression of plasma membrane proteins. J. Membr. Biol. 117, 201–221 (1990). 2231695 10.1007/BF01868451

[b5] Lee H., Lin M. C., Kornblum H. I., Papazian D. M. & Nelson S. F. Exome sequencing identifies *de novo* gain of function missense mutation in *KCND2* in identical twins with autism and seizures that slows potassium channel inactivation. Hum. Mol. Genet. 23, 3481–3489, 10.1093/hmg/ddu056 (2014). 24501278 PMC4049306

[b6] Simons C. . Mutations in the voltage-gated potassium channel gene *KCNH1* cause Temple-Baraitser syndrome and epilepsy. Nat. Genet. 47, 73–77, 10.1038/ng.3153 (2015). 25420144

[b7] Diochot S. . Black mamba venom peptides target acid-sensing ion channels to abolish pain. Nature 490, 552–555, 10.1038/nature11494 (2012). 23034652

[b8] Jensen J. E. . Understanding the molecular basis of toxin promiscuity: The analgesic sea anemone peptide APETx2 interacts with acid-sensing ion channel 3 and hERG channels via overlapping pharmacophores. J. Med. Chem. 57, 9195–9203, 10.1021/jm501400p (2014). 25337890

[b9] Abu-Daya A., Khokha M. K. & Zimmerman L. B. The hitchhiker’s guide to *Xenopus* genetics. Genesis 50, 164–175, 10.1002/dvg.22007 (2012). 22344745 PMC3312310

[b10] Bertin A. . Cellular and molecular characterization of a novel primary osteoblast culture from the vertebrate model organism *Xenopus tropicalis*. Histochem. Cell Biol. 10.1007/s00418-014-1289-8 (2014). 25371327

[b11] de Robertis E. M. & Black P. Hybrids of *Xenopus laevis* and *Xenopus borealis* express proteins from both parents. Dev. Biol. 68, 334–339 (1979). 437325 10.1016/0012-1606(79)90267-7

[b12] Goda T. . Genetic screens for mutations affecting development of *Xenopus tropicalis*. PLoS Genet. 2, e91, 10.1371/journal.pgen.0020091 (2006). 16789825 PMC1475704

[b13] Li B., Russell S. C., Zhang J., Hedrick J. L. & Lebrilla C. B. Structure determination by MALDI-IRMPD mass spectrometry and exoglycosidase digestions of O-linked oligosaccharides from *Xenopus borealis* egg jelly. Glycobiology 21, 877–894, 10.1093/glycob/cwr003 (2011). 21220250 PMC3110487

[b14] Lloyd R. E., Foster P. G., Guille M. & Littlewood D. T. Next generation sequencing and comparative analyses of *Xenopus* mitogenomes. BMC genomics 13, 496, 10.1186/1471-2164-13-496 (2012). 22992290 PMC3546946

[b15] Evans B. J., Kelley D. B., Tinsley R. C., Melnick D. J. & Cannatella D. C. A mitochondrial DNA phylogeny of African clawed frogs: phylogeography and implications for polyploid evolution. Mol. Phylogenet. Evol. 33, 197–213, 10.1016/j.ympev.2004.04.018 (2004). 15324848

[b16] Weber W. M., Liebold K. M., Reifarth F. W. & Clauss W. The Ca^2+^ -induced leak current in *Xenopus* oocytes is indeed mediated through a Cl^-^ channel. J. Membr. Biol. 148, 263–275 (1995). 8747558 10.1007/BF00235044

[b17] Weber W. M., Liebold K. M., Reifarth F. W., Uhr U. & Clauss W. Influence of extracellular Ca^2+^ on endogenous Cl^−^ channels in *Xenopus* oocytes. Pflugers Arch. 429, 820–824 (1995). 7603836 10.1007/BF00374806

[b18] Middleton R. E. . Two tarantula peptides inhibit activation of multiple sodium channels. Biochemistry 41, 14734–14747 (2002). 12475222 10.1021/bi026546a

[b19] Escoubas P. . Isolation of a tarantula toxin specific for a class of proton-gated Na^+^ channels. J. Biol. Chem. 275, 25116–25121, 10.1074/jbc.M003643200 (2000). 10829030

[b20] Diochot S. . A new sea anemone peptide, APETx2, inhibits ASIC3, a major acid-sensitive channel in sensory neurons. EMBO J 23, 1516–1525, 10.1038/sj.emboj.7600177 (2004). 15044953 PMC391081

[b21] Pless S. A. & Lynch J. W. Illuminating the structure and function of Cys-loop receptors. Clin. Exp. Pharmacol. Physiol. 35, 1137–1142, 10.1111/j.1440-1681.2008.04954.x (2008). 18505452

[b22] Larabell C. A. & Chandler D. E. The coelomic envelope of *Xenopus laevis* eggs: a quick-freeze, deep-etch analysis. Dev. Biol. 131, 126–135 (1989). 2909400 10.1016/s0012-1606(89)80044-2

[b23] Solletti J. M., Kasas S., Bertrand D. & Weisenhorn A. L. Atomic force and scanning electron microscopy of *Xenopus laevis* oocytes. J. Vac. Sci. Technol. B 12, 1535–1538, 10.1116/1.587280 (1994).

[b24] Terhag J., Cavara N. A. & Hollmann M. Cave Canalem: how endogenous ion channels may interfere with heterologous expression in *Xenopus* oocytes. Methods 51, 66–74, 10.1016/j.ymeth.2010.01.034 (2010). 20123125

[b25] Aoshima H., Iio H. & Kobayashi S. Li^+^ uptake into *Xenopus* and *Cynops* oocytes injected with exogenous mRNA, observed by flame emission spectroscopy. Anal. Biochem. 156, 257–262 (1986). 3740415 10.1016/0003-2697(86)90181-8

[b26] Kobayashi S., Iio H. & Aoshima H. New translation system of mRNA coding for neurotransmitter receptors using oocytes of the newt. *Cynops pyrrhogaster*. *Brain Res.* 387, 93–96 (1986). 3742236 10.1016/0169-328x(86)90024-0

[b27] Vargas R. A., Botero L., Lagos L. & Camacho M. *Bufo marinus* oocytes as a model for ion channel protein expression and functional characterization for electrophysiological studies. Cell. Physiol. Biochem. 14, 197–202, 10.1159/000080327 (2004). 15319522

[b28] Markovich D. & Regeer R. R. Expression of membrane transporters in cane toad *Bufo marinus* oocytes. J. Exp. Biol. 202, 2217–2223 (1999). 10409492 10.1242/jeb.202.16.2217

[b29] Schroeder B. C., Cheng T., Jan Y. N. & Jan L. Y. Expression cloning of TMEM16A as a calcium-activated chloride channel subunit. Cell 134, 1019–1029, 10.1016/j.cell.2008.09.003 (2008). 18805094 PMC2651354

[b30] Saez N. J. . A dynamic pharmacophore drives the interaction between Psalmotoxin-1 and the putative drug target acid-sensing ion channel 1a. Mol. Pharmacol. 80, 796–808, 10.1124/mol.111.072207 (2011). 21825095

[b31] Schroeder C. I. . Chemical synthesis, 3D structure, and ASIC binding site of the toxin mambalgin-2. Angew. Chem. Int. Ed. Engl. 53, 1017–1020, 10.1002/anie.201308898 (2014). 24323786

[b32] Hall B. J., Chebib M., Hanrahan J. R. & Johnston G. A. 6-Methylflavanone, a more efficacious positive allosteric modulator of gamma-aminobutyric acid (GABA) action at human recombinant α_2_β_2_γ_2L_ than at α_1_β_2_γ_2L_ and α_1_β_2_ GABA_A_ receptors expressed in *Xenopus* oocytes. Eur. J. Pharmacol. 512, 97–104, 10.1016/j.ejphar.2005.02.034 (2005). 15840393

[b33] Wang Q., Pless S. A. & Lynch J. W. Ligand- and subunit-specific conformational changes in the ligand-binding domain and the TM2-TM3 linker of α_1_β_2_γ_2_ GABA_A_ receptors. J. Biol. Chem. 285, 40373–40386, 10.1074/jbc.M110.161513 (2010). 20937799 PMC3001017

[b34] Han L., Talwar S., Wang Q., Shan Q. & Lynch J. W. Phosphorylation of α3 glycine receptors induces a conformational change in the glycine-binding site. ACS Chem. Neurosci 4, 1361–1370, 10.1021/cn400097j (2013). 23834509 PMC3798985

[b35] Twiner M. J. . Marine algal toxin azaspiracid is an open-state blocker of hERG potassium channels. Chem. Res. Toxicol. 25, 1975–1984, 10.1021/tx300283t (2012). 22856456 PMC3444677

[b36] Blanchard M. G., Rash L. D. & Kellenberger S. Inhibition of voltage-gated Na^+^ currents in sensory neurones by the sea anemone toxin APETx2. Br. J. Pharmacol. 165, 2167–2177, 10.1111/j.1476-5381.2011.01674.x (2012). 21943094 PMC3413854

[b37] Pless S. A., Dibas M. I., Lester H. A. & Lynch J. W. Conformational variability of the glycine receptor M2 domain in response to activation by different agonists. J. Biol. Chem. 282, 36057–36067, 10.1074/jbc.M706468200 (2007). 17911099

[b38] Pless S. A. & Lynch J. W. Ligand-specific conformational changes in the α1 glycine receptor ligand-binding domain. J. Biol. Chem. 284, 15847–15856, 10.1074/jbc.M809343200 (2009). 19286654 PMC2708881

[b39] Wang M. H. A technical consideration concerning the removal of oocyte vitelline membranes for patch clamp recording. Biochem. Biophys. Res. Commun. 324, 971–972, 10.1016/j.bbrc.2004.09.162 (2004). 15485648

[b40] Dahan D. S. . A fluorophore attached to nicotinic acetylcholine receptor βM2 detects productive binding of agonist to the αδ site. Proc. Natl. Acad. Sci. USA 101, 10195–10200, 10.1073/pnas.0301885101 (2004). 15218096 PMC454187

[b41] Anangi R., Rash L. D., Mobli M. & King G. F. Functional expression in *Escherichia coli* of the disulfide-rich sea anemone peptide APETx2, a potent blocker of acid-sensing ion channel 3. Mar. Drugs 10, 1605–1618, 10.3390/md10071605 (2012). 22851929 PMC3407934

[b42] Gui J. . A tarantula-venom peptide antagonizes the TRPA1 nociceptor ion channel by binding to the S1-S4 gating domain. Curr. Biol. 24, 473–483, 10.1016/j.cub.2014.01.013 (2014). 24530065 PMC3949122

