# Supplementary material for: Xenopus borealis as an alternative source of oocytes for biophysical and pharmacological studies of neuronal ion channels
Source: Sci Rep. 2015 Oct 6;5:14763. doi: 10.1038/srep14763 (PMC4594035; doi:10.1038/srep14763)
Supplement: Supplementary Information [file srep14763-s1.pdf]

Supplementary Information

Xenopus borealis as an alternative source of oocytes for biophysical and pharmacological studies of neuronal ion channels

Ben Cristofori-Armstrong, Ming S. Soh, Sahil Talwar, Darren L. Brown, John D. O. Griffin, Zoltan Dekan, Jennifer L. Stow, Glenn F. King, Joseph W. Lynch & Lachlan D. Rash

Supplementary Table S1

Comparison of mean values for fast and slow time constants (τ) of deactivation for $K_V10.1$ channels tested in *X. laevis* and *X. borealis* oocytes. There is no evidence of a difference between the two species for all measured parameters (unpaired t-test, $P > 0.05$).

Ion Channel	Tau – deactivation (ms)		*P* value	Reference
$K_V10.1$	*X. laevis*	*X. borealis*		
Slow (–120 mV)	145.9	169.7	0.075	
Slow (–110 mV)	147.6	169.8	0.085	1
Slow (–100 mV)	174.4	193.7	0.257	
Slow (–90 mV)	180.0	198.9	0.187	
Fast (–120 mV)	25.6	27.1	0.725	
Fast (–110 mV)	21.9	25.2	0.406	1
Fast (–100 mV)	22.4	27.3	0.289	
Fast (–90 mV)	20.5	23.9	0.319	

Supplementary Table S2

Properties of MTS-TAMRA labelled α_1N203C and MTSR labelled α_1R271C human GlyRs. Comparison of EC_{50}, slope, maximal current change (ΔI_{max}), and maximal fluorescence change (ΔF_{max}) values tested in *X. laevis* and *X. borealis* oocytes. Data are mean ± s.e.m. There is no evidence of a difference between the two species for all measured parameters (unpaired t-test, $P > 0.05$). Data are comparable to literature values for α_1N203C [2] and α_1R271C [3].

Ion Channel	EC_{50} (μM)		*P* value
GlyR	*X. laevis*	*X. borealis*	
α_1N203C Δ*I*	145 ± 10	136 ± 8.7	0.69
α_1N203C Δ*F*	548 ± 25	408 ± 51	0.63
α_1R271C Δ*I*	1926 ± 175	1698 ± 104	0.52
α_1R271C Δ*F*	872 ± 58	1006 ± 98	0.46
	Slope		*P* value
	X. laevis	*X. borealis*	
α_1N203C Δ*I*	1.9 ± 0.2	1.5 ± 0.1	0.82
α_1N203C Δ*F*	1.4 ± 0.2	1.8 ± 0.1	0.62
α_1R271C Δ*I*	1.4 ± 0.2	2.0 ± 0.2	0.1
α_1R271C Δ*F*	1.3 ± 0.2	1.0 ± 0.1	0.46
	ΔI_{max} (μA) or ΔF_{max} (%)		*P* value
	X. laevis	*X. borealis*	
α_1N203C Δ*I*	8.4 ± 0.2	8.0 ± 0.8	0.63
α_1N203C Δ*F*	25.9 ± 4.2	27.8 ± 4.4	0.77
α_1R271C Δ*I*	3.4 ± 1.7	2.7 ± 0.5	0.69
α_1R271C Δ*F*	12.2 ± 2.2	9.0 ± 2.2	0.34

Supplementary Table S3

Comparison of half-maximal response and slope values for ion channels tested in *X. laevis* and *X. borealis* oocytes and references to studies reporting similar values for these parameters. Steady-state desensitisation: SSD; Activation: act. There is no evidence of a difference between the two species for any of the measured parameters (unpaired t-test, $P > 0.05$).

Ion Channel	Half-maximal response		*P* value	Slope		*P* value	Reference
	X. laevis	*X. borealis*		*X. laevis*	*X. borealis*		
Voltage-gated potassium channels							
K_V10.1	19.7 mV	20.2 mV	0.86	20.8	20.6	0.90	[1]
K_V11.1	–24.3 mV	–23.7 mV	0.18	6.98	7.53	0.16	[4]
Voltage-gated sodium channels							
Na_V1.2	–22.5 mV	–22.3 mV	0.61	4.74	4.60	0.84	[5]
Na_V1.5	–33.5 mV	–33.8 mV	0.49	4.81	4.22	0.08	[6]
Na_V1.7	–14.2 mV	–14.8 mV	0.09	5.24	4.88	0.27	[7]
Acid-sensing ion channels							
ASIC1a pH_{act}	pH 6.11	pH 6.06	0.27	1.95	1.86	0.83	[8]
ASIC1a pH_{SSD}	pH 7.19	pH 7.19	0.66	10.39	9.97	0.69	[8]
ASIC1b pH_{act}	pH 5.95	pH 5.97	0.11	3.98	3.36	0.12	[9]
ASIC1b pH_{SSD}	pH 6.78	pH 6.78	0.72	4.17	4.09	0.91	[10]
ASIC2a pH_{act}	Ambiguous fits with sigmoidal non-linear regression model						
ASIC2a pH_{SSD}	pH 5.99	pH 5.98	0.66	2.27	2.37	0.78	[11]
ASIC3 pH_{act}	pH 6.16	pH 6.12	0.13	1.46	1.60	0.40	[9]
ASIC3 pH_{SSD}	pH 7.04	pH 7.02	0.19	8.25	9.04	0.70	[12]
Pc1a:ASIC1a	1.00 nM	1.03 nM	0.63	1.44	1.60	0.34	[13]
APETx2:ASIC3	64.7 nM	51.1 nM	0.05	0.84	0.93	0.37	[14]
$GABA_A$ receptors							
$\alpha_1\beta_2\gamma_{2L}$	25.1 µM	17.9 µM	0.27	0.56	0.60	0.71	[15]
$\alpha_5\beta_2\gamma_{2L}$	5.18 µM	4.71 µM	0.84	0.49	0.74	0.16	[16]
$\alpha_5\beta_3\gamma_{2L}$	3.10 µM	3.92 µM	0.35	0.75	0.93	0.27	[17]

Supplementary Figure S1

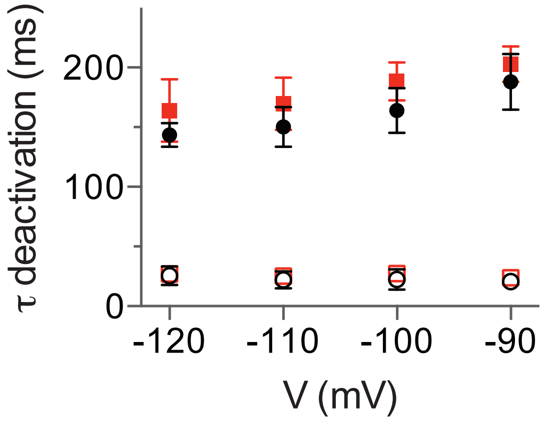

Figure. S1. Deactivation kinetics of Kv10.1 channels expressed in *X. laevis* (●) and *X. borealis* (■) oocytes. Fast (open symbols) and slow (closed symbols) time constants (τ) of deactivation (analysed from the tail current at different voltages following a maximally activating pre-pulse to +80 mV) (n = 12–15). Error bars indicate 95% confidence intervals.

Supplementary References

1 Simons, C. *et al.* Mutations in the voltage-gated potassium channel gene *KCNH1* cause Temple-Baraitser syndrome and epilepsy. *Nat. Genet.* **47**, 73-77, doi:10.1038/ng.3153 (2015).

2 Pless, S. A. & Lynch, J. W. Ligand-specific conformational changes in the α1 glycine receptor ligand-binding domain. *J. Biol. Chem.* **284**, 15847-15856, doi:10.1074/jbc.M809343200 (2009).

3 Pless, S. A., Dibas, M. I., Lester, H. A. & Lynch, J. W. Conformational variability of the glycine receptor M2 domain in response to activation by different agonists. *J. Biol. Chem.* **282**, 36057-36067, doi:10.1074/jbc.M706468200 (2007).

4 Twiner, M. J. *et al.* Marine algal toxin azaspiracid is an open-state blocker of hERG potassium channels. *Chem. Res. Toxicol.* **25**, 1975-1984, doi:10.1021/tx300283t (2012).

5 Nguyen, H. M. & Goldin, A. L. Sodium channel carboxyl-terminal residue regulates fast inactivation. *J. Biol. Chem.* **285**, 9077-9089, doi:10.1074/jbc.M109.054940 (2010).

6 Jones, D. K., Peters, C. H., Tolhurst, S. A., Claydon, T. W. & Ruben, P. C. Extracellular proton modulation of the cardiac voltage-gated sodium channel, $Na_V1.5$. *Biophys. J.* **101**, 2147-2156, doi:10.1016/j.bpj.2011.08.056 (2011).

7 Zhang, M. M. *et al.* Co-expression of $Na_V\beta$ subunits alters the kinetics of inhibition of voltage-gated sodium channels by pore-blocking μ-conotoxins. *Br. J. Pharmacol.* **168**, 1597-1610, doi:10.1111/bph.12051 (2013).
8 Schroeder, C. I. *et al.* Chemical synthesis, 3D structure, and ASIC binding site of the toxin mambalgin-2. *Angew. Chem. Int. Ed. Engl.* **53**, 1017-1020, doi:10.1002/anie.201308898 (2014).
9 Hesselager, M., Timmermann, D. B. & Ahring, P. K. pH Dependency and desensitization kinetics of heterologously expressed combinations of acid-sensing ion channel subunits. *J. Biol. Chem.* **279**, 11006-11015, doi:10.1074/jbc.M313507200 (2004).
10 Chen, X., Kalbacher, H. & Gründer, S. Interaction of acid-sensing ion channel (ASIC) 1 with the tarantula toxin psalmotoxin 1 is state dependent. *J. Gen. Physiol.* **127**, 267-276, doi:10.1085/jgp.200509409 (2006).
11 Salinas, M. *et al.* Binding site and inhibitory mechanism of the mambalgin-2 pain-relieving peptide on acid-sensing ion channel 1a. *J. Biol. Chem.* **289**, 13363-13373, doi:10.1074/jbc.M114.561076 (2014).
12 Yagi, J., Wenk, H. N., Naves, L. A. & McCleskey, E. W. Sustained currents through ASIC3 ion channels at the modest pH changes that occur during myocardial ischemia. *Circ. Res.* **99**, 501-509, doi:10.1161/01.RES.0000238388.79295.4c (2006).
13 Escoubas, P. *et al.* Isolation of a tarantula toxin specific for a class of proton-gated Na^+ channels. *J. Biol. Chem.* **275**, 25116-25121, doi:10.1074/jbc.M003643200 (2000).
14 Diochot, S. *et al.* A new sea anemone peptide, APETx2, inhibits ASIC3, a major acid-sensitive channel in sensory neurons. *EMBO J.* **23**, 1516-1525, doi:10.1038/sj.emboj.7600177 (2004).
15 Hall, B. J., Chebib, M., Hanrahan, J. R. & Johnston, G. A. 6-Methylflavanone, a more efficacious positive allosteric modulator of gamma-aminobutyric acid (GABA) action at human recombinant $\alpha_2\beta_2\gamma_{2L}$ than at $\alpha_1\beta_2\gamma_{2L}$ and $\alpha_1\beta_2$ $GABA_A$ receptors expressed in *Xenopus* oocytes. *Eur. J. Pharmacol.* **512**, 97-104, doi:10.1016/j.ejphar.2005.02.034 (2005).
16 Karim, N. *et al.* Potency of GABA at human recombinant $GABA_A$ receptors expressed in *Xenopus* oocytes: a mini review. *Amino acids* **44**, 1139-1149, doi:10.1007/s00726-012-1456-y (2013).
17 Ramerstorfer, J., Furtmuller, R., Vogel, E., Huck, S. & Sieghart, W. The point mutation γ2F77I changes the potency and efficacy of benzodiazepine site ligands in different $GABA_A$ receptor subtypes. *Eur. J. Pharmacol.* **636**, 18-27, doi:10.1016/j.ejphar.2010.03.015 (2010).
